# Computed Tomographic Study of the Normal Nasal Cavity and Paranasal Sinuses in Persian Cats

**DOI:** 10.1155/vmi/2383627

**Published:** 2026-11-15

**Authors:** Negin Mohammadpour, Siamak Alizadeh, Mohammadreza Hosseinchi

**Affiliations:** ^1^ Faculty of Veterinary Medicine Ur.C. Islamic Azad University Urmia Iran azad.ac.ir; ^2^ Department of Clinical Sciences Faculty of Veterinary Medicine Nag.C. Islamic Azad University Naghadeh Iran azad.ac.ir; ^3^ Department of Basic Sciences Faculty of Veterinary Medicine Ur.C. Islamic Azad University Urmia Iran azad.ac.ir

**Keywords:** computed tomography, diagnostic interpretation, nasal cavities, paranasal, Persian cat, sinuses

## Abstract

**Background:**

Persian cats are predisposed to respiratory disorders due to brachycephalic skull conformation. This study aimed to evaluate the nasal cavity and paranasal sinuses using computed tomography (CT).

**Methodology:**

This study included 10 Persian cats (5 males, 5 females) with a mean age of 3.83 ± 1.18 years and body weight of 4.92 ± 0.81 kg. CT was performed after anesthesia with medetomidine, ketamine, and butorphanol from the nasal tip to the caudal margin of the foramen magnum.

**Results:**

The frontal sinus was the largest paranasal sinus and was divided into four compartments: rostral, medial, lateral, and caudal. The ethmoidal turbinates were located in the caudal nasal cavity and extended into the ventral aspect of the frontal sinus. The maxillary sinus formed a diverticulum of the nasal cavity, with the root of the carnassial tooth projecting into it. The lacrimal, palatine, incisive, nasal, maxillary, and presphenoid bones were best visualized in transverse CT images, whereas the ethmoid and vomer bones were more clearly identified in sagittal images. The zygomatic bone and cribriform plate were best evaluated in dorsal images. The palatine fissures appeared as two oval openings in the cranial hard palate.

**Conclusions:**

These findings provide CT reference data for the nasal cavity and paranasal sinuses in Persian cats, supporting anatomical assessment, clinical image interpretation, and future comparative studies.

## 1. Introduction

Persian cats are characterized by a brachycephalic craniofacial conformation, which results in marked shortening of the facial skeleton and alterations in the anatomical configuration of the upper respiratory tract [1, 2]. In these cats, a greater degree of brachycephaly has been associated with reduced upper airway dimensions, highlighting the clinical relevance of breed‐related craniofacial morphology [3]. This conformation has also been associated with respiratory abnormalities in brachycephalic cats, including narrowing of the nasal passages and increased respiratory signs [4].

Detailed knowledge of the normal sinonasal anatomy is important for accurate interpretation of imaging findings and recognition of pathological changes. The nasal cavity and paranasal sinuses contain complex anatomical structures, including the nasal conchae, ethmoturbinates, nasal septum, and interconnected air‐filled spaces, which contribute to respiratory air conditioning and olfaction [5]. Computed tomography (CT) provides detailed cross‐sectional and three‐dimensional evaluation of these structures and is therefore particularly useful for assessing normal anatomy and sinonasal abnormalities in cats [6].

Previous CT studies have described characteristic findings in feline sinonasal diseases. Tromblee et al. reported ethmoturbinate, maxillary, vomer, and orbital lamina lysis, together with abnormal soft tissue or fluid attenuation in the sphenoid and frontal sinuses and retrobulbar space in cats with paranasal sinus neoplasia [7]. Barrs et al. identified nasal cavity involvement, paranasal sinus soft tissue attenuation, orbital and turbinate lysis, and mass lesions in feline upper respiratory aspergillosis [4]. Likewise, Detweiler et al. found bulla effusion to be a common CT feature of inflammatory and neoplastic sinonasal disease, with nasopharyngeal lymphoma being the neoplasm most frequently associated with this finding [8].

Oliveira et al. reported that, in feline nasopharyngeal polyps, inflammatory lesions appeared on CT as well‐defined pedunculated masses and were frequently associated with asymmetric thickening of the tympanic bulla wall [9]. Furthermore, Finck et al., in a comparative study of CT and rhinoscopy for the evaluation of suspected feline nasal neoplasia, concluded that CT was more reliable than rhinoscopy for the initial detection of nasal tumors and may therefore be considered a first‐line diagnostic modality [6].

Despite the clinical relevance of brachycephalic conformation in Persian cats, detailed CT‐based descriptions and morphometric reference data for the normal nasal cavity and paranasal sinuses in this breed remain limited. Characterizing these structures may improve the interpretation of sinonasal imaging findings and help distinguish normal anatomical variation from pathological changes. Therefore, this study aimed to describe the CT anatomy and morphometric characteristics of the nasal cavity, nasal septum, and paranasal sinuses in clinically healthy adult Persian cats and to establish preliminary reference data for these structures.

## 2. Materials and Methods

### 2.1. Study Design and Animals

This prospective descriptive cross‐sectional study included 10 adult Persian cats (five males and five females) selected from three Persian cat breeding and rearing centers. All cats were owned by these centers and had a mean age of 3.83 ± 1.18 years and a mean body weight of 4.92 ± 0.81 kg (Table 1). Prior to inclusion, all cats underwent veterinary clinical and general physical examinations, assessment of respiratory signs, and oral and dental examinations, with no clinical evidence of respiratory, sinonasal, or dental disease. Cats with relevant clinical abnormalities were excluded. Although the chronological ages of the cats were available from the center records, conventional radiographic examination and dental formula assessment were performed to confirm adult developmental status [10]. All cats were maintained under appropriate nutritional conditions.

**TABLE 1:** Weight and age of studied Persian cats.

Cats	Male						Female						Total mean ± SD
	1	2	3	4	5	Mean ± SD	1	2	3	4	5	Mean ± SD	
Weight (kg)	4.7	5.4	6.3	5.7	5.4	5.5 ± 0.57	3.8	4.3	4.7	4.6	4.3	4.34 ± 0.35	4.92 ± 0.81
Age (years)	3.5	4.6	2.5	3	5.5	3.82 ± 1.14	2	3.5	5.5	3.6	4.6	3.84 ± 1.21	3.83 ± 1.18

### 2.2. Anesthesia Protocol

The cats used in the study were placed under general anesthesia using dissociative agents. For this purpose, a combination of medetomidine HCl (0.03–0.05 mg/kg, IM, Medetate 0.1% injectable, Jorux, England), ketamine HCl (7–10 mg/kg, IM, Ketasol 10% injectable, Alfasan, Holland), and butorphanol tartrate (0.4 mg/kg, Butomidor 1% injectable, Richter Pharma, Austria) was administered via the intramuscular route [11].

### 2.3. CT Equipment and Procedure

Each cat was positioned in sternal recumbency with the head extended forward and carefully aligned to minimize rotation. The hard palate and mandibular axis were positioned perpendicular to the gantry to ensure symmetrical acquisition of the cranial structures. A CT examination was performed from the tip of the nose to the caudal margin of the foramen magnum using a helical multislice CT scanner (Asteion Premium 4, Toshiba Medical Systems, Model TSX‐021B, and Japan). Scanning was performed using a tube voltage of 120 kVp, a tube current of 50 mAs, a gantry rotation time of 400 ms, and a pitch of 1. Images were acquired with a slice thickness of 1 mm. The acquisition parameters also included a physical detector collimation of 32 × 0.6 mm and a final section collimation of 64 × 0.6 mm. A 512 × 512 matrix was used, providing an in‐plane spatial resolution of approximately 0.92 × 0.92 mm [12]. The acquired CT datasets were subsequently reconstructed at 0.5‐mm intervals and exported in Digital Imaging and Communications in Medicine (DICOM) format for further image reconstruction, morphological evaluation, and morphometric analysis.

### 2.4. Three‐Dimensional Reconstruction

The acquired CT datasets were stored in DICOM format and transferred to a dedicated workstation for three‐dimensional image reconstruction and analysis using Onis CT software (Multi‐Modality Workplace, Version 2.5A) [13]. Transverse images were used as the primary dataset, and sagittal and dorsal multiplanar reformatted images were generated from the reconstructed volumetric dataset to facilitate three‐dimensional assessment of the nasal cavity and paranasal sinuses. For anatomical evaluation and three‐dimensional reconstruction, bone window settings (window width, 4000 HU; window level, 550 HU) were used to optimize visualization of the osseous structures and air‐filled paranasal sinuses, whereas soft‐tissue window settings (window width, 450 HU; window level, 80 HU) were used when evaluation of soft‐tissue structures was required. The reconstructed datasets were subsequently used for morphological and morphometric assessment of the paranasal sinuses, nasal cavity, and nasal septum.

### 2.5. Morphological and Morphometric Analyses

Morphological and morphometric evaluations were performed using the reconstructed CT datasets in transverse, sagittal, and dorsal planes. The transverse images served as the primary dataset, while sagittal and dorsal multiplanar reformatted images were used to facilitate visualization of anatomical structures that were more clearly delineated in specific planes. Morphological assessment focused on the identification, configuration, symmetry, spatial extent, and anatomical relationships of the nasal cavity, nasal septum, nasal conchae and meatuses, ethmoidal turbinates, and the frontal, maxillary, sphenoidal, and ethmoidal sinuses. Particular attention was given to the subdivision and communication of the frontal sinus, the relationship of the maxillary sinus to the nasal cavity and adjacent teeth, and the extension and spatial arrangement of the ethmoidal turbinates. The adjacent osseous structures were also evaluated based on their CT visibility and anatomical relationships.

Morphometric measurements were obtained from the reconstructed CT images using the electronic caliper and region‐based measurement tools available in Onis CT software (Multi‐Modality Workplace, Version 2.5A). To ensure reproducibility, all measurements were performed using predefined anatomical landmarks, standardized measurement planes, consistent caliper orientation, and identical measurement criteria for all animals. The same anatomical boundaries and measurement procedures were applied independently by both observers. Linear measurements of the paranasal sinuses included maximum length and maximum width. Sinus length was defined as the greatest rostrocaudal distance between the most rostral and most caudal identifiable boundaries of each sinus, measured along its principal rostrocaudal axis on the reconstructed plane showing the maximum longitudinal extent. Sinus width was defined as the greatest mediolateral distance between the medial and lateral boundaries of the same sinus on the corresponding transverse or multiplanar reformatted image. The same measurement definitions and procedures were applied consistently to the frontal, maxillary, sphenoidal, and ethmoidal sinuses. For the nasal cavity, length was measured along the rostrocaudal axis from the most rostral identifiable entrance of the nasal cavity to the caudal choanal region. Nasal cavity width was defined as the maximum mediolateral distance between the lateral boundaries of the nasal cavity, whereas nasal cavity height was defined as the maximum dorsoventral distance between its dorsal and ventral boundaries. Measurements were obtained on the reconstructed CT plane that provided the clearest visualization of the corresponding anatomical boundaries, and the same measurement orientation was maintained for all animals. Nasal septum length was measured from its rostral to caudal extent. Septal thickness was measured separately at the cranial and caudal portions of the nasal septum, corresponding to the cranial and caudal septal thickness parameters reported in the morphometric analysis.

Three‐dimensional volumetric measurements of the frontal, maxillary, sphenoidal, and ethmoidal sinuses and the nasal cavity were obtained by manual region‐of‐interest (ROI) segmentation of consecutive reconstructed CT images using Onis CT software. The boundaries of each structure were delineated slice by slice according to its identifiable anatomical margins, excluding adjacent air‐containing spaces whenever distinguishable. Nasal cavity segmentation extended from the rostral nasal opening to the caudal choanae. The software calculated the volume of each structure from the segmented regions [14].

All morphometric measurements were performed independently by two observers using the same predefined anatomical landmarks, measurement planes, and segmentation criteria. The measurements were then averaged for each animal and morphometric parameter, and the resulting mean values were used in the subsequent statistical analyses. All anatomical structures and measurements were identified and described according to the Nomina Anatomica Veterinaria [15].

### 2.6. Statistical Analysis

Statistical analyses were performed using IBM SPSS Statistics Version 26 (IBM Corp., Armonk, NY, USA), and graphs were generated using GraphPad Prism Version 9 (GraphPad Software, San Diego, CA, USA). Morphometric data were summarized as mean ± standard deviation (SD). Distributional assumptions were assessed using the Shapiro–Wilk test and visual inspection. Sex‐related differences were evaluated using Welch’s independent‐samples *t*‐test or the Mann–Whitney *U* test, as appropriate. All tests were two‐sided. *p* values for sex comparisons were adjusted using the Holm procedure across the prespecified family of morphometric variables, and both unadjusted and adjusted *p* values were reported.

Associations between morphometric variables were assessed using Pearson’s correlation coefficient (*r*). The 78 unique pairwise correlations were adjusted using the Benjamini–Hochberg procedure, with significance evaluated using adjusted *q*‐values. Four simple linear regression models, each including an intercept and one explanatory variable, were fitted to characterize selected associations. Results included *r*, *R* ^2^, standardized (*β*), the unstandardized slope with its 95% confidence interval (CI), and the corresponding *p* value. Regression *p* values were adjusted using the Holm procedure. Given the small sample size, correlation and regression analyses were considered exploratory and hypothesis‐generating, not confirmatory or predictive.

All measurements were independently obtained by two observers using predefined anatomical landmarks, imaging planes, and criteria. Intraobserver reliability was assessed by repeating measurements after 2 weeks, with the observer blinded to the initial results. Interobserver and intraobserver reliability were evaluated using the intraclass correlation coefficient (ICC), based on a two‐way mixed‐effects model, absolute agreement, and single measurements. ICC estimates were reported with 95% CIs; values ≥ 0.90 were interpreted as excellent reliability and values of 0.75–0.89 as good reliability. Reliability results are presented in Table 2.

**TABLE 2:** Interobserver and intraobserver reliability of CT‐based morphometric measurements in Persian cats.

Morphometric parameter	Interobserver ICC	95% CI	Intraobserver ICC	95% CI
Frontal sinus volume (cm^3^)	0.96	0.91–0.99	0.98	0.95–0.99
Maxillary sinus volume (cm^3^)	0.94	0.87–0.98	0.97	0.93–0.99
Sphenoidal sinus volume (cm^3^)	0.91	0.80–0.97	0.95	0.88–0.98
Ethmoidal sinus volume (cm^3^)	0.93	0.85–0.98	0.96	0.91–0.99
Nasal cavity volume (cm^3^)	0.95	0.89–0.98	0.97	0.93–0.99
Frontal sinus length (mm)	0.97	0.93–0.99	0.98	0.95–0.99
Frontal sinus width (mm)	0.95	0.89–0.98	0.97	0.93–0.99
Maxillary sinus length (mm)	0.96	0.91–0.99	0.98	0.95–0.99
Maxillary sinus width (mm)	0.94	0.87–0.98	0.97	0.93–0.99
Sphenoidal sinus length (mm)	0.92	0.82–0.97	0.95	0.88–0.98
Sphenoidal sinus width (mm)	0.90	0.77–0.96	0.94	0.86–0.98
Ethmoidal sinus length (mm)	0.93	0.85–0.98	0.96	0.91–0.99
Ethmoidal sinus width (mm)	0.91	0.80–0.97	0.95	0.88–0.98
Nasal cavity length (mm)	0.98	0.95–0.99	0.99	0.97–1.00
Nasal cavity width (mm)	0.96	0.91–0.99	0.98	0.95–0.99
Nasal cavity height (mm)	0.95	0.89–0.98	0.97	0.93–0.99
Nasal septum length (mm)	0.97	0.93–0.99	0.98	0.95–0.99
Cranial septal thickness (mm)	0.92	0.82–0.97	0.95	0.88–0.98
Caudal septal thickness (mm)	0.90	0.77–0.96	0.94	0.86–0.98

*Note:* ICCs represent single‐measure absolute agreement using a two‐way mixed‐effects model [ICC(3,1)]. Interobserver reliability was assessed between two independent observers; intraobserver reliability was assessed using repeated measurements by the same observer. All ICCs are reported with 95% CIs; higher ICCs indicate greater reliability.

Abbreviations: CI, confidence interval; ICC, intraclass correlation coefficient.

## 3. Results

The lacrimal, palatine, incisive, nasal, maxillary, and presphenoid bones were more clearly delineated in the transverse plane (Figure 1). In addition, the ethmoid and vomer bones were best visualized in the sagittal plane (Figure 2A), whereas the zygomatic bone was more readily identified in the dorsal plane (Figure 2B). These osseous structures extended from the external nares to the cribriform plate of the ethmoid bone and the nasopharynx. The cribriform plate was best evaluated in the dorsal plane, where it appeared as a sieve‐like structure located between the nasal and cranial cavities and composed of numerous small foramina (Figure 2B).

**FIGURE 1:**
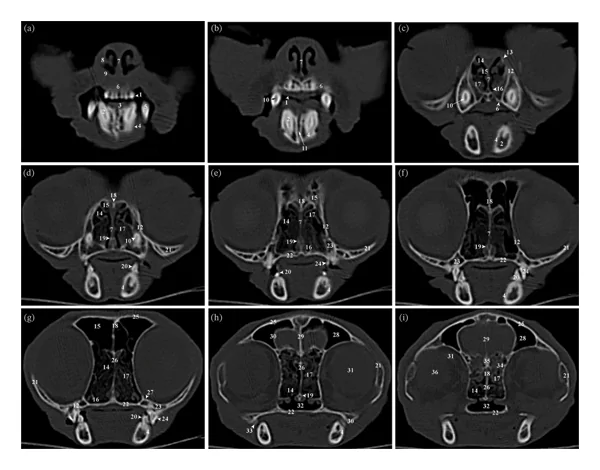
(A). CT scan image (longitudinal view—bone window) and (a–i). Transverse computed tomography reconstruction images of the normal head of a 3‐year‐old male Persian cat. 1. Maxillary incisors 2. Root of mandibular canine tooth 3. Mandibular incisors 4. Mandibular body 5. Maxillary canine 6. Incisive bone 7. Nasal cartilaginous septum 8. Caudal portion of the alar fold (dorsal part) 9. Caudal portion of the alar fold (ventral part) 10. Root of maxillary canine tooth 11. Symphysis of mandible 12. Maxilla bone 13. Nasal bone 14. Nasal cavity 15. Dorsal nasal meatus 16. Ventral nasal meatus 17. Ethmoid turbinates 18. Osseous nasal septum (dorsal portion) 19. Vomer bone 20. Mandibular premolar tooth 21. Zygomatic bone 22. Hard palate 23. Infraorbital canal 24. Maxillary premolar teeth 25. Frontal bone 26. Osseous nasal septum (ventral portion) 27. Lacrimal bone 28. Rostral portion of frontal sinus 29. Medial septum of frontal sinus 30. Caudal portion of maxilla 31. Eye orbit 32. Nasopharynx 33. Molar teeth of the maxillary 34. Cribriform plate of ethmoid bone 35. Olfactory bulb 36. Lens.

**FIGURE 2:**
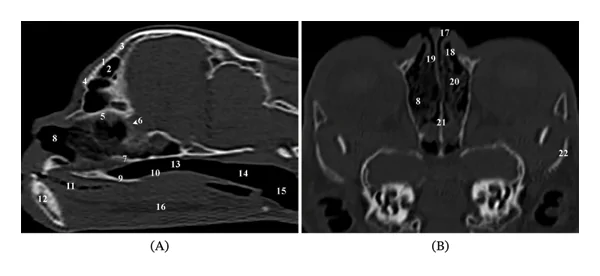
Sagittal (A) and dorsal (B) reformatted computed tomographic images of the same Persian cat head. 1. Frontal bone 2. Frontal sinus 3. Squamous part of frontal bone 4. Nasal part of frontal bone 5. Ethmoid bone 6. Cribriform plate of ethmoid bone 7. Vomer bone 8. Nasal cavity 9. Hard palate 10. Soft palate 11. Oral cavity 12. Root of mandibular canine tooth 13. Nasal part of pharynx 14. Pharynx 15. Laryngeal part of pharynx 16. Tongue 17. Dorsal nasal meatus 18. Middle nasal meatus 19. Common nasal meatus 20. Ethmoid labyrinth 21. Osseous nasal septum 22. Zygomatic bone.

The right and left nasal cavities were separated by a nasal septum, which was cartilaginous and membranous rostrally and osseous caudally. The ethmoidal turbinates were located in the caudal nasal cavity, attached to the cribriform plate and covered by the orbital lamina. They extended ventrally into the frontal sinus and consisted of four endoturbinates and six ectoturbinates. The dorsal and ventral nasal conchae were identified in the rostral nasal cavity. The dorsal concha arose from the nasal bone, whereas the thicker, shorter ventral concha originated from the maxilla, occupied approximately two‐thirds of the rostral nasal cavity, and extended rostrally with the alar fold. Four nasal meatuses were identified: dorsal, middle, ventral, and common. The common nasal meatus, located between the nasal septum and conchae, communicated with the other three meatuses (Figure 1).

The nasopharyngeal meatus extended from the caudal ventral nasal meatus to the choana and opened into the nasopharynx. CT images clearly identified the frontal sinus, the bilateral maxillary sinuses, and the nasal fundus, all in continuity with the nasal cavity (Figures 1 and 2). The frontal sinus was the largest paranasal sinus, communicated with the nasal cavity, and was subdivided into four compartments: rostral (*sinus frontalis rostralis*), medial (*sinus frontalis medialis*), lateral (*sinus frontalis lateralis*), and caudal (*sinus frontalis caudalis*). The palatine fissures appeared as two oval openings in the cranial hard palate, immediately caudal to the incisor teeth (Figure 1). The maxillary sinus formed a diverticulum of the nasal cavity and appeared as a distinct recess in one female cat. The roots of the fourth premolar (carnassial) tooth projected into this sinus. The sphenoidal sinus occupied a substantial portion of the sphenoid bone, whereas the ethmoidal sinus was smaller and located between the neurocranial and facial regions of the skull (Figure 2A).

As shown in Table 3, the frontal sinus had the greatest volume and width, whereas the maxillary sinus had the greatest length in both sexes. Male cats had numerically larger sinus volumes and dimensions than females. However, after Holm adjustment, only the sex difference in sphenoidal sinus width (*p* = 0.010) and ethmoidal sinus length (*p* = 0.030) remained statistically significant. Although frontal and maxillary sinus volumes were numerically greater in males, neither sex difference remained statistically significant after Holm adjustment. Overall, all paranasal sinus measurements were greater in males, whereas the sphenoidal sinus showed the least individual variation. As shown in Table 4, nasal cavity width was significantly greater in males than females after Holm adjustment (*p* = 0.048). Although males showed numerically greater values for the other nasal cavity and septal measurements, these differences were not statistically significant after adjustment (Figure 3).

**TABLE 3:** Computed tomography measurements of paranasal sinus volumes (cm^3^) and linear dimensions (width and length; mm) in male and female Persian cats.

Variable		Male (*n* = 5)	Female (*n* = 5)	Total (*n* = 10)	*p* value	Holm‐adjusted *p* value
Sinus volume (cm^3^)	Frontal	2.91 ± 0.23	2.43 ± 0.25	2.67 ± 0.34	0.013	0.166
	Maxillary	1.91 ± 0.23	1.57 ± 0.13	1.74 ± 0.25	0.024	0.205
	Sphenoid	0.24 ± 0.01	0.21 ± 0.01	0.23 ± 0.01	0.009	0.134
	Ethmoid	0.41 ± 0.03	0.39 ± 0.01	0.40 ± 0.02	0.429	0.895

Sinus width (mm)	Frontal	22.13 ± 1.27	19.54 ± 0.30	20.83 ± 1.62	0.009	0.134
	Maxillary	7.55 ± 0.29	6.95 ± 0.06	7.25 ± 0.37	0.009	0.134
	Sphenoid	4.38 ± 0.15	3.82 ± 0.16	4.10 ± 0.32	0.001	0.010
	Ethmoid	5.38 ± 0.23	4.96 ± 0.23	5.17 ± 0.31	0.022	0.205

Sinus length (mm)	Frontal	14.09 ± 0.41	13.39 ± 0.15	13.74 ± 0.47	0.018	0.205
	Maxillary	19.17 ± 0.50	18.43 ± 0.33	18.80 ± 0.55	0.029	0.207
	Sphenoid	9.28 ± 0.37	8.33 ± 0.36	8.80 ± 0.61	0.003	0.056
	Ethmoid	12.35 ± 0.45	11.11 ± 0.21	11.73 ± 0.73	0.001	0.030

*Note:* Values are reported as mean ± standard deviation (SD). Between‐sex comparisons were performed using Welch’s independent‐samples *t*‐test. Unadjusted and Holm‐adjusted *p* values are presented, with significance defined as Holm‐adjusted *p* < 0.05.

**TABLE 4:** Computed tomography measurements of nasal cavity and septum parameters in male and female Persian cats (in mm).

Parameter		Male (*n* = 5)	Female (*n* = 5)	Total (*n* = 10)	*p* value	Holm‐adjusted *p* value
Nasal cavity	Length	29.72 ± 1.91	27.47 ± 0.72	28.59 ± 1.80	0.056	0.281
	Width	18.77 ± 0.83	16.55 ± 0.24	17.66 ± 1.30	0.002	0.048
	Height	14.41 ± 0.88	12.98 ± 0.28	13.69 ± 0.97	0.019	0.205

Nasal septum	Length	30.09 ± 1.09	29.18 ± 0.24	29.63 ± 0.88	0.138	0.554
	Cr thickness	1.44 ± 0.15	1.36 ± 0.05	1.40 ± 0.11	0.325	0.895
	Ca thickness	1.92 ± 0.12	1.73 ± 0.03	1.82 ± 0.13	0.030	0.207

*Note:* Values are presented as mean ± standard deviation (SD). Between‐sex comparisons used Welch’s independent‐samples *t*‐test. Holm‐adjusted *p* < 0.05 was considered statistically significant. Cr, cranial; Ca, caudal.

**FIGURE 3:**
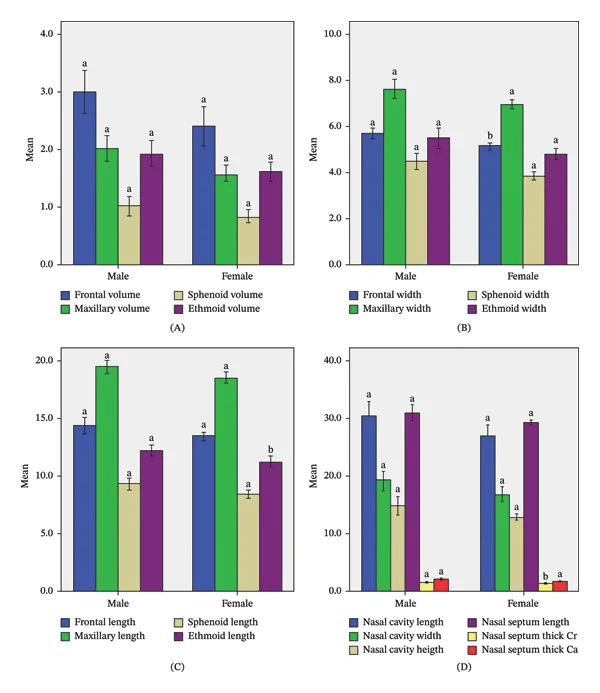
Comparison of paranasal sinus volume (A; cm^3^), width (B; mm), and length (C; mm), and linear measurements of the nasal cavity and nasal septum (D; mm) between male and female Persian cats. Within each column, means with different superscript letters (a, b) indicate statistically significant differences between the two groups (*p* < 0.05).

Exploratory Pearson correlation analysis identified several positive associations among paranasal sinus, nasal cavity, and nasal septum measurements (Table 5). Strong correlations were observed between frontal sinus volume and length (*r* = 0.91), maxillary sinus volume and length (*r* = 0.96), and nasal cavity height and caudal septal thickness (*r* = 0.98). These findings should be interpreted cautiously because of the small sample size and the exploratory nature of the analysis.

**TABLE 5:** Pearson correlation coefficient among morphometric parameters of the paranasal sinuses, nasal cavity, and nasal septum in Persian cats.

Variable	FSV	MSV	SSV	ESV	FSL	MSL	NCV	NCL	NCW	NCH	NSL	NST‐Cr	NST‐Ca
FSV	1.00	0.89^∗∗^	0.85^∗∗^	0.56	0.91^∗∗^	0.79^∗^	0.63	0.90^∗∗^	0.85^∗∗^	0.88^∗∗^	0.67	0.60	0.81^∗^
MSV	0.89^∗∗^	1.00	0.77^∗^	0.57	0.80^∗^	0.96^∗∗^	0.61	0.90^∗∗^	0.85^∗∗^	0.83^∗∗^	0.53	0.45	0.74^∗^
SSV	0.85^∗∗^	0.77^∗^	1.00	0.62	0.78^∗^	0.63	0.31	0.77^∗^	0.80^∗^	0.64	0.41	0.36	0.60
ESV	0.56	0.57	0.62	1.00	0.62	0.50	0.63	0.73^∗^	0.56	0.51	0.32	0.52	0.56
FSL	0.91^∗∗^	0.80^∗^	0.78^∗^	0.62	1.00	0.76^∗^	0.77^∗^	0.93^∗∗^	0.93^∗∗^	0.94^∗∗^	0.83^∗∗^	0.79^∗^	0.95^∗∗^
MSL	0.79^∗^	0.96^∗∗^	0.63	0.50	0.76^∗^	1.00	0.65	0.85^∗∗^	0.83^∗∗^	0.84^∗∗^	0.57	0.49	0.75^∗^
NCV	0.63	0.61	0.31	0.63	0.77^∗^	0.65	1.00	0.82^∗^	0.62	0.78^∗^	0.72^∗^	0.79^∗^	0.83^∗∗^
NCL	0.90^∗∗^	0.90^∗∗^	0.77^∗^	0.73^∗^	0.93^∗∗^	0.85^∗∗^	0.82^∗^	1.00	0.87^∗∗^	0.89^∗∗^	0.70^∗^	0.69^∗^	0.88^∗∗^
NCW	0.85^∗∗^	0.85^∗∗^	0.80^∗^	0.56	0.93^∗∗^	0.83^∗∗^	0.62	0.87^∗∗^	1.00	0.93^∗∗^	0.68^∗^	0.60	0.91^∗∗^
NCH	0.88^∗∗^	0.83^∗∗^	0.64	0.51	0.94^∗∗^	0.84^∗∗^	0.78^∗^	0.89^∗∗^	0.93^∗∗^	1.00	0.84^∗∗^	0.76^∗^	0.98^∗∗^
NSL	0.67	0.53	0.41	0.32	0.83^∗∗^	0.57	0.72^∗^	0.70^∗^	0.68^∗^	0.84^∗∗^	1.00	0.94^∗∗^	0.87^∗∗^
NST‐Cr	0.60	0.45	0.36	0.52	0.79^∗^	0.49	0.79^∗^	0.69^∗^	0.60	0.76^∗^	0.94^∗∗^	1.00	0.81^∗^
NST‐Ca	0.81^∗^	0.74^∗^	0.60	0.56	0.95^∗∗^	0.75^∗^	0.83^∗∗^	0.88^∗∗^	0.91^∗∗^	0.98^∗∗^	0.87^∗∗^	0.81^∗^	1.00

*Note:* Values represent Pearson’s correlation coefficients (*r*). The 78 unique pairwise correlations were adjusted using the Benjamini–Hochberg procedure. Significance was defined as adjusted (*q* < 0.05) (^∗^) and (*q* < 0.01) (^∗∗^). NST‐Cr, cranial nasal septum thickness; NST‐Ca, caudal nasal septum thickness.

Abbreviations: ESV, ethmoid sinus volume; FSL, frontal sinus length; FSV, frontal sinus volume; MSL, maxillary sinus length; MSV, maxillary sinus volume; NCH, nasal cavity height; NCL, nasal cavity length; NCV, nasal cavity volume; NCW, nasal cavity width; NSL, nasal septum length; SSV, sphenoid sinus volume.

Exploratory simple linear regression analyses identified significant associations between frontal sinus length and volume (*R* ^2^ = 0.825, adjusted *p* < 0.001), maxillary sinus length and volume (*R* ^2^ = 0.925, adjusted *p* < 0.001), nasal cavity height and volume (*R* ^2^ = 0.611, adjusted *p* = 0.008), and nasal cavity width and length (*R* ^2^ = 0.753, adjusted *p* = 0.002) (Table 6). These associations describe covariation within the present sample and should not be interpreted as validated predictive relationships. Measurement reliability was high across all morphometric parameters. Interobserver ICCs ranged from 0.90 to 0.98, whereas intraobserver ICCs ranged from 0.94 to 0.99 (Table 2), indicating generally excellent reliability, although some CIs were relatively wide.

**TABLE 6:** Exploratory simple linear regression analyses of selected morphometric associations in Persian cats.

Dependent variable (Y)	Independent variable (X)	Pearson’s r	*R* ^2^	Standardized *β*	Unstandardized slope (95% CI)	*p* value	Holm‐adjusted *p* value
Frontal sinus volume (cm^3^)	Frontal sinus length (mm)	0.908	0.825	0.908	0.645 (0.403–0.887)	< 0.001	< 0.001
Maxillary sinus volume (cm^3^)	Maxillary sinus length (mm)	0.962	0.925	0.962	0.441 (0.338–0.543)	< 0.001	< 0.001
Nasal cavity volume (cm^3^)	Nasal cavity height (mm)	0.781	0.611	0.781	0.814 (0.284–1.344)	0.008	0.008
Nasal cavity length (mm)	Nasal cavity width (mm)	0.868	0.753	0.868	1.203 (0.640–1.765)	0.001	0.002

*Note:* r, Pearson’s correlation coefficient; *R* ^2^, coefficient of determination; β, standardized coefficient; slope, unstandardized coefficient; CI, confidence interval (95% CI for the slope). *p* values are two‐sided tests of a zero slope. Holm adjustment was applied across the four tests; all associations remained significant.

## 4. Discussion

Radiography remains the most widely used imaging modality for evaluating cranial diseases in small animals, particularly dogs and cats, owing to its availability, low cost, and ease of use [16]. However, the complex anatomy of the skull and superimposition of osseous and soft tissue structures often limit lesion detection, especially in the nasal cavity, paranasal sinuses, and other intricate cranial regions. Consequently, radiography may not reliably identify structural alterations, inflammatory lesions, neoplasia, osseous destruction, or changes associated with chronic respiratory disease. Advances in veterinary diagnostic imaging have increased the use of cross‐sectional modalities that provide superior visualization of anatomical relationships and occult lesions [17]. These techniques improve diagnostic accuracy and support treatment planning, prognosis, and therapeutic monitoring, particularly in anatomically complex regions such as the skull and paranasal sinuses.

CT is an important imaging modality for evaluating the nasal cavity and paranasal sinuses in veterinary medicine [18]. Its cross‐sectional and multiplanar capabilities reduce tissue superimposition and facilitate the assessment of osseous structures, air‐filled cavities, and anatomical relationships. Three‐dimensional reconstruction further supports morphological and morphometric evaluation and may assist clinical interpretation and surgical planning. Previous CT studies in several animal species, including cats, have demonstrated its value for investigating cranial and sinonasal anatomy [19–25]. In the present study, CT enabled detailed assessment of the nasal cavity, nasal conchae, meatuses, nasal septum, and paranasal sinuses in Persian cats.

One of the notable findings of this study was the distinctive morphology of the ventral nasal concha. It was thick, short, and occupied approximately two‐thirds of the rostral nasal cavity. Originating from the maxilla, it appeared as an extension of the second endoturbinate, forming multiple elongated scrolls that increased the mucosal surface area for warming, humidifying, and filtering inspired air. These observations agree with Schlueter et al., who described the ventral nasal concha as a large, well‐developed structure involved in airflow regulation [5]. Similarly, Ragab et al. reported its rostral extension in domestic cats [26]. In the present study, the ventral concha extended rostrally in association with the alar fold. This finding may be associated with the brachycephalic craniofacial conformation of Persian cats; however, its potential influence on airflow and airway resistance requires further functional investigation. Given the recognized clinical importance of upper‐airway disorders in brachycephalic feline conformation, this anatomical configuration may be particularly relevant when the nasal lumen is compromised by mucosal inflammation, edema, or space‐occupying lesions. The relatively large and rostrally extended ventral nasal concha may reduce the remaining luminal space under such pathological conditions and thereby potentially increase the functional impact of nasal obstruction. These findings provide useful reference data for CT interpretation and differentiation of normal anatomy from pathological changes.

Wu et al. reported that the ethmoid turbinates in domestic cats are mainly confined to the caudal nasal cavity [27]. In contrast, the present study showed that in Persian cats they also extended into the ventral aspect of the frontal sinus, suggesting a relatively extensive ethmoturbinate complex in the examined Persian cats. This finding may be associated with the brachycephalic cranial conformation; however, a breed‐specific association cannot be established from the present sample. The observed extension of the ethmoturbinates may represent a morphological feature of the nasal cavity, but its effects on olfactory mucosal surface area and nasal airflow cannot be determined from the present CT‐based morphometric data and should therefore be considered hypotheses for future functional studies. In the presence of mucosal swelling, inflammatory changes, or space‐occupying lesions, the prominent intranasal turbinate structures could theoretically further narrow the available nasal lumen; however, any resulting effect on airflow or the functional severity of nasal obstruction was not assessed in the present study. Clinically, recognition of this normal anatomical feature is important for accurate CT interpretation and for distinguishing normal anatomical complexity from pathological changes associated with chronic rhinitis, fungal sinonasal disease, sinusitis, nasal polyps, or neoplasia. Because this feature has received limited attention previously, it may represent a characteristic morphological trait of Persian cats; however, its breed specificity and potential functional effects require further comparative and functional investigation among feline breeds.

In the present study, the cribriform plate of the ethmoid bone was clearly identified on CT images, particularly in the dorsal plane. This delicate, sieve‐like structure forms the boundary between the nasal and cranial cavities and contains multiple foramina transmitting olfactory nerve fibers to the olfactory bulb. Its clear visualization highlights the value of CT for assessing complex nasal anatomy. Clinically, accurate evaluation of the cribriform plate is essential because invasive nasal neoplasms, fungal infections, and destructive ethmoidal lesions may extend intracranially through this structure. The location and morphology of the cribriform plate were consistent with previous descriptions in other felids, suggesting that despite breed‐specific differences in the nasal cavity and ethmoid turbinates, the cribriform plate remains highly conserved among felid species [28].

The present study confirmed that the frontal sinus is the largest paranasal sinus in Persian cats, occupies much of the frontal air space, and communicates directly with the nasal cavity. It comprises four compartments: rostral (*sinus frontalis rostralis*), medial (*sinus frontalis medialis*), lateral (*sinus frontalis lateralis*), and caudal (*sinus frontalis caudalis*). This configuration agrees with previous reports in carnivores [29, 30]. Unlike dogs, particularly dolichocephalic breeds in which the frontal sinus may extend into adjacent cranial bones, the frontal sinus in Persian cats remains largely confined to the frontal bone, reflecting their brachycephalic skull conformation. The large air‐filled frontal sinus is an important anatomical feature of the examined cats; although paranasal sinuses have been proposed to have structural and functional roles, such effects were not evaluated in the present study and should therefore not be inferred from the observed CT morphology. These findings further characterize the morphology of the frontal sinus in the examined Persian cats and may contribute to understanding anatomical variation associated with brachycephalic cranial conformation.

The present study showed that the maxillary sinus in Persian cats appears as a distinct depression within the maxillary bone and can be regarded as a diverticulum of the nasal cavity, consistent with Karnik et al. [31]. Although the term “maxillary recess” has been used in some anatomical descriptions of the domestic cat, the term “maxillary sinus” is retained in the present study for consistency with the anatomical structure evaluated and measured on CT. CT images demonstrated that the roots of the fourth premolar (carnassial) tooth were closely associated with, and occasionally projected into, the maxillary sinus. This close spatial relationship may be particularly relevant to the interpretation of sinonasal disease in Persian cats because the limited maxillary space and close proximity of the carnassial tooth root may facilitate clinical overlap between dental and sinonasal abnormalities. Accordingly, dental disease should be considered when focal maxillary sinus or adjacent nasal abnormalities are identified on CT, particularly when inflammatory sinonasal changes are present. This anatomical relationship represents a clinically testable hypothesis that should be investigated in Persian cats with naturally occurring dental and sinonasal disease. Accordingly, detailed knowledge of the relationship between the maxillary sinus and caudal maxillary dentition is essential for CT interpretation and surgical or dental planning. Compared with the frontal sinus, the maxillary sinus was less developed, likely reflecting the limited facial space of the brachycephalic skull. Nevertheless, its close association with the nasal cavity and carnassial teeth makes it a key structure in diseases affecting the nasal and maxillary regions.

Previous studies have described the sphenoidal sinus in domestic cats as relatively small and confined to the sphenoid bone [32]. In contrast, the sphenoidal sinus appeared relatively extensive in the examined Persian cats. This finding may be related to differences in cranial conformation, although confirmation of a breed‐specific association requires comparative studies involving other feline breeds. Because of its close proximity to the pituitary gland, optic chiasm, and brain base, accurate knowledge of its anatomy is important for CT interpretation and clinical procedures. Conversely, the ethmoid sinus was relatively small and located between the facial and parietal bones, consistent with Abouelela et al. and Proctor and Chang [33, 34]. Its broadly similar morphology across the felid species described in previous studies may indicate relative morphological stability; however, evolutionary conservation and the influence of breed‐related cranial variation cannot be established from the present study.

The number, morphology, and arrangement of the nasal conchae and meatuses were consistent with previous anatomical descriptions of felids. Each nasal cavity contained dorsal and ventral nasal conchae and ethmoid turbinates, forming a complex airway that increases the mucosal surface for warming, humidifying, filtering inspired air, and directing airflow toward the olfactory region. The observed configuration of the nasal cavity may have functional and clinical implications in Persian cats. The relatively prominent nasal conchae and ethmoturbinate structures, together with the compact cranial conformation observed in these cats, may be relevant to the available nasal lumen; however, whether this anatomical configuration limits airway reserve or alters airflow cannot be determined from the present morphometric data. This anatomical relationship provides a plausible basis for further investigation of the association between sinonasal morphology and upper‐airway disorders, particularly chronic rhinitis, inflammatory sinonasal disease, and obstructive nasal lesions in Persian cats. However, because airflow dynamics and disease prevalence were not evaluated in the present study, these interpretations should be regarded as hypotheses rather than demonstrated causal relationships. Environmental and management factors may also influence upper respiratory morphology; therefore, the observed differences probably result from interactions between genetic and environmental factors rather than breed alone.

Male Persian cats generally showed numerically greater paranasal sinus measurements than females. However, after Holm adjustment, significant sex differences were observed only in sphenoidal sinus width and ethmoidal sinus length. These findings should be interpreted cautiously in light of previous reports of craniofacial variation in cats [35]. These differences likely reflect the greater cranial dimensions of males and should be considered when interpreting sinus morphology and diagnosing pathological changes. In contrast, the sphenoidal sinus showed the least morphometric variation, consistent with Curtis et al., who reported limited individual variation in this sinus among small mammals [36]. Its deep location and close association with vital neural structures may be relevant to its relative morphological stability; however, the developmental or evolutionary mechanisms underlying this stability were not evaluated in the present study.

Although males showed numerically greater nasal cavity and septal measurements, only nasal cavity width remained significantly different between sexes after Holm adjustment. The observed differences may inform preliminary morphometric reference values, but their functional implications remain uncertain because airflow and respiratory function were not assessed.

However, the present study did not directly assess airflow dynamics or respiratory function; therefore, the functional implications of these dimensional differences remain uncertain. Previous reports have suggested associations between nasal dimensions and airway resistance or airflow dynamics [37], but whether similar relationships exist in Persian cats remains a hypothesis requiring direct functional assessment. Both nasal septum length and thickness were greater in male Persian cats. Because the septum separates the nasal cavities and contributes to the structural framework of the nose, these differences may be relevant when interpreting CT findings and establishing sex‐specific reference values. However, the functional consequences of the observed differences were not evaluated in this study.

Positive correlations were observed among several paranasal sinus, nasal cavity, and nasal septum measurements (Table 5). The strongest associations included frontal sinus volume with length (*r* = 0.91) and maxillary sinus volume with length (*r* = 0.96). These relationships indicate covariation within the examined sample but do not establish shared developmental mechanisms or functional consequences.

Exploratory regression analysis revealed strong associations between frontal and maxillary sinus length and volume. Associations were also observed between nasal cavity height and volume, and between nasal cavity width and length. These findings are consistent with Martínez et al., who investigated feline sinonasal anatomy using advanced imaging techniques [38]. The observed correlations indicate morphometric covariation, but their functional and clinical implications remain uncertain because airflow and diseased cats were not assessed. These findings provide preliminary reference data for future comparative studies of healthy and diseased Persian cats; however, the observed associations should not be interpreted as validated predictive models for sinus volume or disease.

An important methodological finding was the high reproducibility of the CT‐based morphometric measurements. All parameters showed excellent interobserver and intraobserver reliability, supporting the consistency of the predefined anatomical landmarks, measurement procedures, and CT reconstruction protocol. These findings strengthen the value of the reported measurements as preliminary reference data for future anatomical and comparative studies in Persian cats.

The anatomical findings of this study suggest several testable hypotheses regarding upper‐airway disease in Persian cats. The relatively compact nasal cavity and prominent nasal conchal and ethmoturbinate structures may limit the available airway reserve and potentially increase the functional consequences of mucosal inflammation or space‐occupying lesions. The extension of the ethmoturbinates into the ventral aspect of the frontal sinus may also be relevant when distinguishing normal anatomical variation from pathological changes associated with chronic rhinitis, fungal disease, sinusitis, or sinonasal neoplasia. In addition, the close relationship between the maxillary sinus and carnassial tooth root provides an anatomical basis for investigating interactions between dental and sinonasal disease. These proposed associations should not be interpreted as evidence of causation but rather as clinically relevant hypotheses generated from the present morphometric findings and warranting confirmation through comparative CT, airflow, endoscopic, and clinical studies.

Despite its anatomical and clinical value, the present study should be considered a preliminary descriptive investigation of sinonasal anatomy and paranasal sinus morphometry in Persian cats. The relatively small sample size (*n* = 10) may have reduced statistical power and limits the generalizability of the findings. In addition, because only healthy adult Persian cats were included, age‐related changes and pathological conditions could not be evaluated. In particular, the absence of a normocephalic control group prevents determination of whether the observed nasal cavity and turbinate configuration differs quantitatively from that of cats with a non‐brachycephalic cranial conformation. Therefore, the potential relationship between the observed anatomy and upper‐airway disease should be considered hypothesis‐generating rather than confirmatory. Therefore, the morphological and morphometric findings reported here should be interpreted cautiously and require confirmation in larger feline populations and comparative studies involving different breeds. Such studies may help determine whether the observed anatomical features are specifically associated with Persian breed characteristics or reflect broader variations in feline craniofacial conformation.

Although CT provides excellent assessment of osseous structures and air‐filled cavities, it is less suitable for evaluating soft tissues and microscopic changes. Future studies combining CT with MRI or micro‐CT may therefore provide more comprehensive anatomical information. Studies involving larger sample sizes and a wider range of age groups could further characterize the development and age‐related changes of the paranasal sinuses and nasal cavity and contribute to the establishment of age‐specific reference values. Future comparative studies should specifically evaluate whether the reduced or compact nasal dimensions and prominent turbinate configuration identified in Persian cats are associated with measurable differences in nasal airflow resistance. Combining CT‐based morphometry with computational airflow modeling, endoscopy, and clinical assessment of cats with upper‐airway disease would help determine whether these anatomical features represent true risk factors for respiratory disease or simply breed‐associated morphological variation. In addition, correlating CT findings with clinical examinations, endoscopy, histopathology, and surgical observations could enhance the clinical relevance of morphometric parameters and support the development of more reliable criteria for the assessment of nasal and paranasal sinus disorders. Such studies would further strengthen the anatomical and clinical understanding of the sinonasal region in Persian cats and other felid species.

## 5. Conclusion

This study provides preliminary CT‐based anatomical and morphometric reference data for the nasal cavity and paranasal sinuses of adult Persian cats. The frontal sinus was the largest paranasal sinus and comprised four compartments, while the ethmoidal turbinates extended into its ventral aspect. The maxillary sinus formed a diverticulum of the nasal cavity and was closely associated with the root of the carnassial tooth. After Holm adjustment, significant sex differences were identified only in sphenoidal sinus width, ethmoidal sinus length, and nasal cavity width. Other morphometric measurements were numerically greater in males but did not remain statistically significant after adjustment. However, these findings should be interpreted cautiously because of the small sample size (*n* = 10) and the absence of comparative data from other feline breeds. The observed morphometric correlations and regression relationships are exploratory and require validation in larger studies. Further comparative research is needed to establish the generalizability and breed specificity of these anatomical findings.

## Funding

No funding was received for this manuscript.

## Disclosure

All authors have read and approved the final version of the manuscript. The corresponding author had full access to all of the data in this study and takes complete responsibility for the integrity of the data and the accuracy of the data analysis.

The corresponding author affirms that this manuscript is an honest, accurate, and transparent account of the study being reported; that no important aspects of the study have been omitted; and that any discrepancies from the study as planned have been explained.

## Ethics Statement

Approval for this study was granted by the Ethical Review Committee of the Islamic Azad University, Urmia Branch, Urmia, Iran (Approval Number: IR.IAU.SRB.REC.1403.086), ensuring compliance with ethical standards for research involving animals. The experiment was conducted under the supervision of the Iranian Society for the Prevention of Cruelty to Animals, emphasizing the commitment to humane treatment throughout the study. The ethical approval document is available online at https://ethics.research.ac.ir/form/oa8v70sixymecp4.pdf.

## Conflicts of Interest

The authors declare no conflicts of interest.

## Data Availability

The data that support the findings of this study are available on request from the corresponding author. The data are not publicly available due to privacy or ethical restrictions.
